# Transcriptional profiling of defense responses to *Botrytis cinerea* infection in leaves of *Fragaria vesca* plants soil-drenched with β-aminobutyric acid

**DOI:** 10.3389/fpls.2022.1025422

**Published:** 2022-12-08

**Authors:** Raghuram Badmi, Torstein Tengs, May Bente Brurberg, Abdelhameed Elameen, Yupeng Zhang, Lisa Karine Haugland, Carl Gunnar Fossdal, Timo Hytönen, Paal Krokene, Tage Thorstensen

**Affiliations:** ^1^ Division of Biotechnology and Plant Health, Norwegian Institute of Bioeconomy Research, Ås, Norway; ^2^ Department of Plant Sciences, Norwegian University of Life Sciences (NMBU), Ås, Norway; ^3^ Department of Agricultural Sciences, University of Helsinki, Helsinki, Finland; ^4^ Organismal and Evolutionary Biology Research Programme, Faculty of Biological and Environmental Sciences, University of Helsinki, Helsinki, Finland; ^5^ Department of Genetics, Genomics and Breeding, National Institute of Agricultural Botany- East Malling Research Station, East Malling, United Kingdom

**Keywords:** strawberry, β-aminobutyric acid, *Botrytis cinerea*, crop protection, disease resistance, necrotroph, transcriptomics

## Abstract

Grey mold caused by the necrotrophic fungal pathogen *Botrytis cinerea* can affect leaves, flowers, and berries of strawberry, causing severe pre- and postharvest damage. The defense elicitor β-aminobutyric acid (BABA) is reported to induce resistance against *B. cinerea* and many other pathogens in several crop plants. Surprisingly, BABA soil drench of woodland strawberry (*Fragaria vesca*) plants two days before *B. cinerea* inoculation caused increased infection in leaf tissues, suggesting that BABA induce systemic susceptibility in *F. vesca*. To understand the molecular mechanisms involved in *B. cinerea* susceptibility in leaves of *F. vesca* plants soil drenched with BABA, we used RNA sequencing to characterize the transcriptional reprogramming 24 h post-inoculation. The number of differentially expressed genes (DEGs) in infected *vs*. uninfected leaf tissue in BABA-treated plants was 5205 (2237 upregulated and 2968 downregulated). Upregulated genes were involved in pathogen recognition, defense response signaling, and biosynthesis of secondary metabolites (terpenoid and phenylpropanoid pathways), while downregulated genes were involved in photosynthesis and response to auxin. In control plants not treated with BABA, we found a total of 5300 DEGs (2461 upregulated and 2839 downregulated) after infection. Most of these corresponded to those in infected leaves of BABA-treated plants but a small subset of DEGs, including genes involved in ‘response to biologic stimulus‘, ‘photosynthesis‘ and ‘chlorophyll biosynthesis and metabolism’, differed significantly between treatments and could play a role in the induced susceptibility of BABA-treated plants.

## Introduction

Cultivated strawberry (*Fragaria × ananassa*) is a commercially important soft fruit crop producing berries rich in beneficial vitamins, antioxidants, and nutrients. However, this crop is exposed to malignant viruses, nematodes, fungi and bacteria that can cause severe yield losses (Garrido et al., 2011). The ascomycete fungus *Botrytis cinerea* is a broad-spectrum necrotrophic pathogen causing severe damage in more than 1000 plant species, including >200 crop species (Williamson et al., 2007; Fillinger and Elad, 2016). *Botrytis cinerea* can cause grey mold by infecting the berries, flowers and leaves of strawberry and is one of the most economically important pathogens in this crop. Strawberry susceptibility towards *B. cinerea* varies with tissue type, ripening stage of berries, and leaf age (Meng et al., 2019; Petrasch et al., 2019).

Plants have a standing defense consisting of constitutive physical and chemical barriers such as cell walls, a waxy cuticle, trichomes, and phytoanticipins (Piasecka et al., 2015). Plants also possess inducible defenses that are activated in response to attack or damage. In the first line of inducible defense, pattern-triggered immunity (PTI) is activated when pattern recognition receptors (PRRs) on the plant cell membrane recognize and bind to specific microbe-, pathogen- or damage-associated molecular patterns (MAMPs, PAMPs, and DAMPs, respectively). Pathogens that successfully evade PTI by secreting pathogen effectors can in turn be recognized by intracellular nucleotide-binding leucine-rich-repeat (NB-LRR) proteins, encoded by R-genes. R-proteins/NB-LRR-proteins activate the second line of inducible defense, known as effector-triggered immunity (ETI).

Activation of PTI and ETI may result in local defense response at the site of pathogen attack, as well as systemic defense responses in more distal tissues. Local defense responses are associated with mitogen-activated protein kinase (MPK) signaling, release of reactive oxygen species, expression of PR-proteins, cell-wall modification, phytohormone synthesis and signaling, and accumulation of secondary metabolites with antimicrobial activity such as phytoalexins (Zeilinger et al., 2016; AbuQamar et al., 2017; Wan et al., 2021). Localized infection or perception of MAMPs/PAMPs/DAMPs may also sensitize both local and more distant tissues for enhanced defense following a subsequent challenge in a process known as defense priming (Amil-Ruiz et al., 2011; Conrath et al., 2015; Reimer-Michalski and Conrath, 2016).

Defense priming is established through a signaling cascade where elicitors, consisting of natural or synthetic substances, are recognized by PRRs that in turn activate the innate immune system. Synthetic elicitors may be analogues of defense or signaling molecules, like acibenzolar-S-methyl (BTH/Bion), β-aminobutyric acid (BABA), and methyl jasmonate (MeJA), or natural compounds of biological origin, such as chitosan, laminarin, and substances produced by beneficial bacteria (Wiesel et al., 2014; Bektas and Eulgem, 2015; Tripathi et al., 2019). Chitosan and MeJA have been shown to increase post-harvest resistance to *B. cinerea* infection in berries when strawberry plants were sprayed before harvesting (Saavedra et al., 2017).

The non-proteinogenic amino acid BABA is an extensively studied defense elicitor. It has been reported to protect some 40 plant species against >80 pests and pathogens (Cohen, 2002; Cohen et al., 2016; Wilkinson et al., 2018) by activating both salicylic acid (SA)-dependent and SA-independent defenses (Ton and Jakab, 2005). BABA can be synthesized chemically for use in crop protection, but is also produced in low concentrations by stressed plants (Thevenet et al., 2017). Studies have shown that BABA induces local and systemic resistance against *B. cinerea* infection in several plant species, including grape, cucumber, Arabidopsis, grapefruit, tomato and strawberry (Cohen et al., 2016; Martínez-Aguilar et al., 2016; Wilkinson et al., 2018). In strawberry, most studies so far have focused on local effects of BABA in berries. Immersing detached berries in a BABA solution provided dose-dependent protection against *B. cinerea* infection. High BABA concentrations induced direct defense responses whereas low concentrations induced defense priming, with full activation of plant defenses only occurring upon subsequent *B. cinerea* infection (Wang et al., 2016). Large-scale transcriptional profiling of strawberry berries infected with *B. cinerea* has previously identified differential expression of genes involved in pathogen recognition, synthesis of secondary metabolites, signaling transduction, defense responses, and cell transport (Xiong et al., 2018; Haile et al., 2019).

With the aim to identify local and systemic resistance effects of BABA we compared responses to *B. cinerea* infection in *F. vesca* leaves on plants sprayed directly with BABA, plants subjected to soil drench with BABA, and untreated control plants. We did a global transcriptomic analysis of leaf tissue using RNA-seq to understand (1) the molecular underpinning of responses to *B. cinerea* infection in untreated plants and (2) systemic effects of BABA soil drench on *B. cinerea*-mediated transcription. Our results reveal strawberry-specific and application method-specific responses to BABA that may be important for the practical use of defense elicitors in future crop protection schemes.

## Results

### *Botrytis cinerea* infection of leaves and effect of BABA treatment on susceptibility

To establish a working quantification method for *B. cinerea* infection and verify pathogenicity, *F. vesca* leaves were drop-inoculated with a spore suspension of *B. cinerea* isolate Bc101 and incubated under high humidity to promote fungal infection. We detected clear symptoms of infection, in the form of necrotic lesions on leaves, 4-5 days after infection (**Supplementary Figure S1**). As expected, qPCR quantification of *B. cinerea* relative to *F. vesca* using genome-specific primers for *B. cinerea* (Bc3F and Bc3R) and *F. vesca* (EF1αF and EF1αR) detected high levels of *B. cinerea* in infected leaves and negligible levels in mock-infected control leaves, indicating fungal disease progression in the necrotic lesions (**Supplementary Figure S1B**).

**Figure 1:**
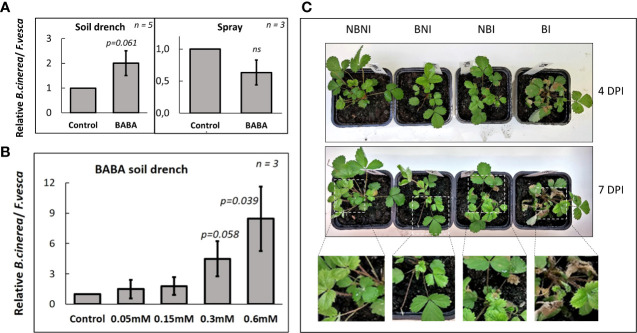
Effects of BABA on *Fragaria vesca* resistance to *Botrytis cinerea* infection of leaves. **(A)** *B. cinerea* infection 5 days post-inoculation in leaves of *F. vesca* plants after soil-drenching (left) with 0.2 mM β-aminobutyric acid (BABA) or foliar spraying with 0.3 mM BABA (right), or **(B)** soil-drenching with 0 mM, 0.05 mM, 0.15 mM, 0.3 mM or 0.6 mM BABA two days before inoculation. Levels of *B. cinerea* infection was measured as the ratio between *B. cinerea* and *F. vesca* DNA using qPCR with genome-specific primers for *B. cinerea* (Bc3F and Bc3R) and *F. vesca* (EF1αF and EF1αR). Levels of *B. cinerea* in untreated control plants are set to 1. ‘*n’* = number of biological replicates (i.e. individual plants), and error bars show standard error ( ± 1 SE). P-values report comparisons between control plants and plants subjected BABA using Student’s t-test. ‘*ns*’ = not statistically significant. **(C)** Representative pictures of disease progression in *F. vesca* plants 4 days and 7 days after inoculation, n = 5. Treatment combinations: *B. cinerea*-infected (NBI; No-BABA and Infected), mock-infected (NBNI; No-BABA and Non-Infected), BABA-treated and *B. cinerea*-infected (BI; BABA and Infected), and BABA-treated and mock-infected (BNI; BABA and Non-Infected).

We then sprayed leaves of *F. vesca* plants with a *B. cinerea* spore suspension (10^6^ spores mL^-1^) until run-off. As for the drop inoculation, necrotic lesions began to appear 4-5 days after spraying. Surprisingly, in *F. vesca* Hawaii-4 plants that had been treated with BABA as a soil drench two days before infection qPCR-quantification showed that *B. cinerea* levels in the leaves were about twice as high as in untreated control plants (p = 0.061; **Figure 1A**). The increased susceptibility to *B. cinerea* after soil drench with BABA corresponded with increased browning and development of larger necrotic lesions (which increased over time) compared to non-treated controls (**Figure 1C**). BABA soil-drench also induced susceptibility to *B. cinerea* infection in *F. vesca* ‘Alexandria’ plants in a dose-dependent manner, with >10-fold increase relative to untreated control in plants treated with 0.6 mM BABA (p = 0.039; **Figure 1B**). The negative effect of BABA on resistance to *B. cinerea* was further confirmed in *F. ananassa*, where plants treated with BABA soil drench suffered larger lesions than untreated controls following *B. cinerea* infection (p = 0.042; **Supplementary Figure S2**). Taken together our observations suggest a systemic negative effect on resistance in leaves when BABA was applied to roots as soil drench.

In contrast, foliar application of BABA two days before infection had a positive, but non-significant, effect on resistance to *B. cinerea* infection relative to control plants that had not been treated with BABA (**Figure 1A**, right), suggesting that locally BABA had a neutral or weakly positive effect on resistance. *Botrytis cinerea* grew normally on potato dextrose agar amended with high concentrations of BABA, suggesting that the local effect observed *in planta* was not due to direct negative effects of BABA on *B. cinerea* growth and survival, but rather was due to induced defense responses (**Supplementary Figure S3**). As defense elicitors may repress plant growth, we investigated potential growth effects on uninfected *F. vesca* plants treated with BABA. Soil drench with BABA significantly inhibited plant growth (**Supplementary Figure S4A**), whereas foliar application did not have any significant effect (**Supplementary Figure S4B**).

### Transcriptional analysis of *Fragaria vesca* leaves infected with *Botrytis cinerea*

RNA-seq was performed to contrast the molecular mechanisms involved in defense against *B. cinerea* in untreated plants and plants treated with 0.2 mM BABA soil-drench two days before infection. We analyzed young leaf samples (n = 5) from plants that had been subjected to four different treatments: *B. cinerea*-infected only (No BABA and Infected; NBI), mock-infected only (No BABA and Non-Infected; NBNI), BABA-treated and *B. cinerea*-infected (BABA and Infected; BI), and BABA-treated and mock-infected (BABA and Non-Infected; BNI). Leaf samples were collected 24 hours after infection (i.e., before any visual symptoms appeared for the infected plants). Illumina sequencing generated an average of 26,176,008 reads per plant (median: 25,291,981) with mapping efficiencies ranging from 75 to 97% (median: 83%). The data were submitted to NCBI’s Sequence Read Archive (SRA) as BioProject PRJNA818508. Reads could be mapped to a total of 28,588 annotated genes in the *F. vesca* genome, and DEGs were identified by pairwise comparisons of gene expression for the different treatments using the R package EdgeR with false discovery rate < 0.05 and absolute log2-fold change values > 1.

Among the differentially expressed genes (DEGs) in non-BABA treated plants infected with *B. cinerea*, 2461 were upregulated and 2839 were downregulated after infection (comparison of NBI *vs*. NBNI). In plants treated with BABA before infection, we identified a total of 5518 DEGs after infection (2483 upregulated and 3035 downregulated in BI *vs*. NBNI and 2237 upregulated and 2968 downregulated in BI *vs*. BNI). A small but significant subset of DEGs were identified for the direct BI *vs.* NBI comparison (9 downregulated and 3 upregulated) and for the BNI *vs.* NBNI comparison (7 downregulated and 4 upregulated) (**Supplementary Table S1**, **S2**).

**Figure 2:**
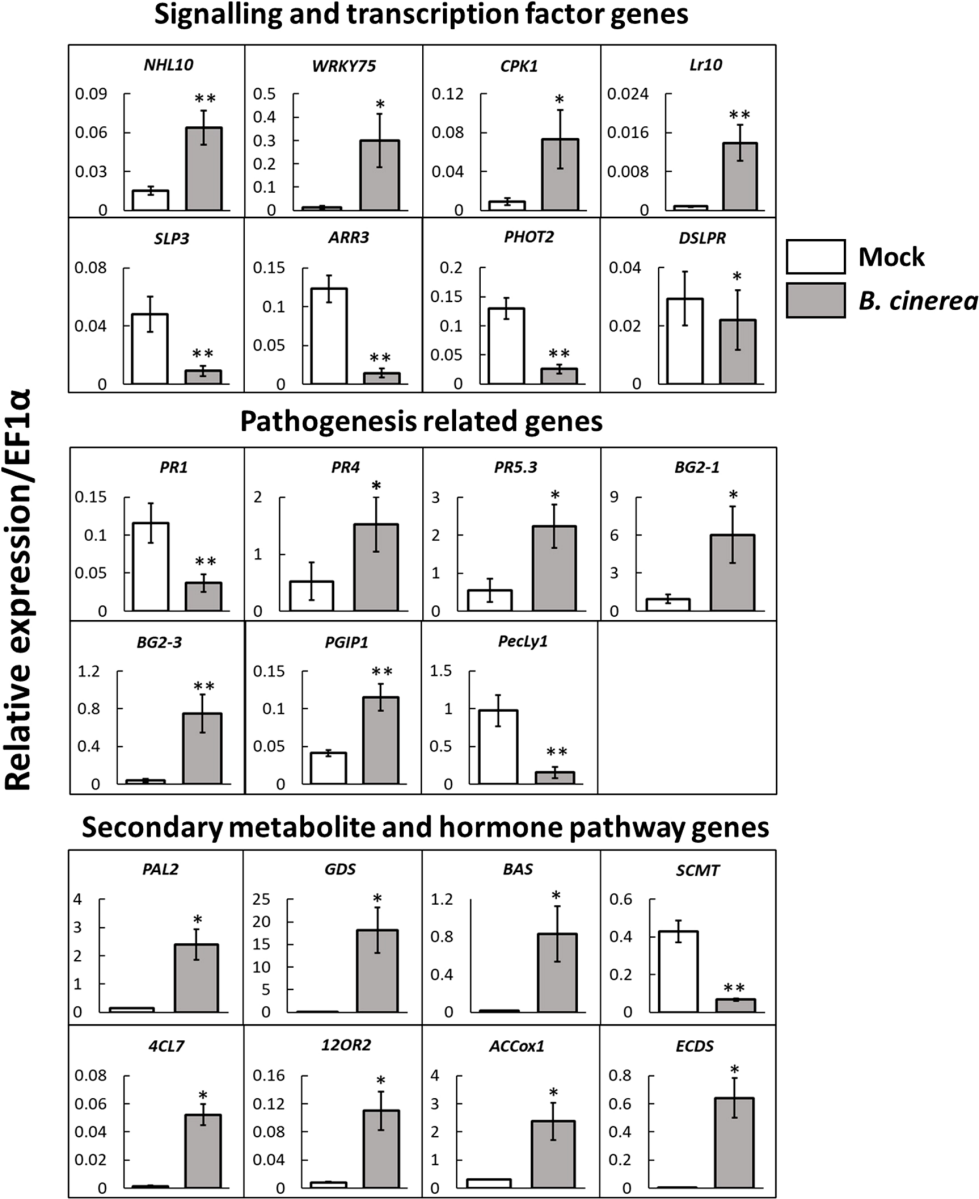
Validation of expression levels by qRT-PCR of selected genes in leaves of *Fragaria vesca* infected with *Botrytis cinerea*. White bars; mock-infected plants, grey bars; plants infected with *B cinerea*. The housekeeping gene *EF1α* was used as an endogenous control gene and expression values were calculated using the ΔCt method. *n = 5* plants per treatment. Error bars show standard error ( ± 1 SE). Student’s t-test: *p < 0.05, **p < 0.01.

qRT-PCR quantification of gene expression of selected DEGs between NBI and NBNI plants showed similar regulation as the RNA-seq data (**Figure 2** and **Table 1**).

**Table 1:** Differentially expressed genes selected for qRT-PCR expression analysis from *Fragaria vesca* leaves infected with *Botrytis cinerea*.

Gene groups	Gene ID (F_vesca_v4.0)	Gene Name	Gene Annotation	log_2_FC	P adjusted
**Signaling and transcription factors:**					
	FvH4_6g20130.1	NHL10	protein YLS9-like	2.333	0.00013
	FvH4_6g53770.1	WRKY75	probable WRKY transcription factor 75	4.957	4.4E-36
	FvH4_6g20840.1	CPK1	transferring phosphorus-containing groups; calcium/calmodulin-dependent protein kinase	3.292	7.8E-18
	FvH4_4g26910.1	Lr10	probable receptor-like protein kinase At1g67000	1.231	0.0012
	FvH4_4g07370.1	SLP3	subtilisin-like protease SBT2.5	-2.602	5.6E-10
	FvH4_4g35230.1	ARR3	two-component response regulator ARR5	-2.919	1.5E-39
	FvH4_2g17220.1	PHOT2	putative LOV domain-containing protein	-2.049	1.7E-16
	FvH4_2g20290.1	DSLPR	WD repeat-containing protein 70		
**Pathogenesis related:**					
	FvH4_2g02920.1	PR1	pathogenesis-related protein 1-like	-1.374	0.0013
	FvH4_3g05950.1	PR4	pathogenesis-related protein PR-4-like	3.887	7.7E-15
	FvH4_6g16950.1	PR5.3	thaumatin-like protein	2.195	3.3E-05
	FvH4_3g28390.1	BG2-1	glucan endo-1,3-beta-glucosidase-like	3.092	6.3E-21
	FvH4_4g19500.1	BG2-3	glucan endo-1,3-beta-glucosidase, basic vacuolar isoform	4.885	5.9E-39
	FvH4_6g22790.1	PGIP1	polygalacturonase-inhibiting protein	1.728	7.2E-11
	FvH4_4g05760.1	PecLy1	probable pectate lyase 8	-4.456	1.9E-26
**Secondary metabolite and hormone pathways:**					
Salicylic acid	FvH4_7g19130.1	PAL2	phenylalanine ammonia-lyase	2.539	4.5E-15
Salicylic acid	FvH4_3g03130.1	SCMT	salicylate carboxymethyltransferase-like	-2.784	3.2E-13
Terpenoids	FvH4_6g11440.1	GDS	(-)-germacrene D synthase-like	6.279	3.1E-48
Terpenoids	FvH4_6g36500.1	BAS	beta-amyrin synthase	3.485	8.2E-07
Flavonoids	FvH4_6g16460.1	4CL7	4-coumarate–CoA ligase-like 7	6.767	3.4E-20
Jasmonic acid	FvH4_5g32630.1	12OR2	putative 12-oxophytodienoate reductase 11	2.773	1.9E-22
Ethylene	FvH4_5g19290.1	ACCox1	1-aminocyclopropane-1-carboxylate oxidase homolog 1-like	2.881	1.0E-32
Gibberellin	FvH4_2g23440.1	ECDS	ent-copalyl diphosphate synthase, chloroplastic-like	6.814	9.4E-55

The genes are grouped after their annotated function according to the *Fragaria vesca* 4.0.a1 annotation. Log_2_-fold change values (Log_2_FC) and adjusted P-values are from the *Botrytis cinerea* infected RNA-seq dataset presented in this publication (**Supplementary Table 1**).

**Figure 3:**
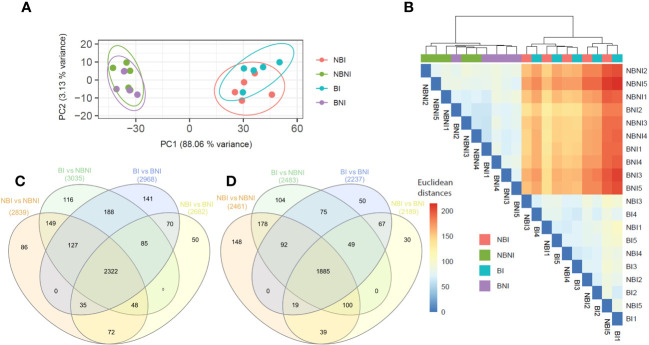
Co-expressed and unique *Fragaria vesca* transcripts after β-aminobutyric acid (BABA) treatment and *Botrytis cinerea* infection. **(A)** Principal component analysis (PCA) analyses of samples based on RNA-Seq data (confidence ellipses with 95% confidence interval). **(B)** Euclidean distances between samples. Analyses were performed using the R package pcaExplorer (version 2.22.0). **(C, D)** Venn diagrams of co-expressed and uniquely expressed genes (Log2 > 1) after pairwise comparison of different treatments: **(C)** downregulated and **(D)** upregulated differentially expressed genes. Treatment combinations: *B. cinerea*-infected (NBI; No-BABA and Infected), mock-infected (NBNI; No-BABA and Non-Infected), BABA-treated and *B. cinerea*-infected (BI; BABA and Infected), and BABA-treated and mock-infected (BNI; BABA and Non-Infected).

Principal component analysis (PCA) was performed to visualize the variability between and within experimental treatments (**Figure 3A**). The first principal component separated infected (BI and NBI) and non-infected (BNI and NBNI) samples, irrespective of BABA-treatment. The fact that the two infected and non-infected treatments grouped together suggests that BABA had little global effect on gene expression. A similar pattern was shown in the cluster dendrogram (**Figure 3B**), where transcriptomes from infected and uninfected samples clustered together, while BABA-treated samples did not cluster.

For a more detailed analysis at the single gene level, we made a Venn diagram to display DEGs from the pairwise treatment comparisons (**Supplementary Table S2**) that were significantly up- or downregulated after BABA treatment and infection (BI) relative to non-infected controls (BNI and/or NBNI). A total of 229 (104 + 75 + 50) genes were upregulated (**Figure 3C**), and 445 (116 + 188 + 141) were downregulated (**Figure 3D** and **Supplementary Table S3**).

### GO enrichment and MAPMAN analysis of DEGs after *Botrytis cinerea* infection and BABA treatment

**Figure 4:**
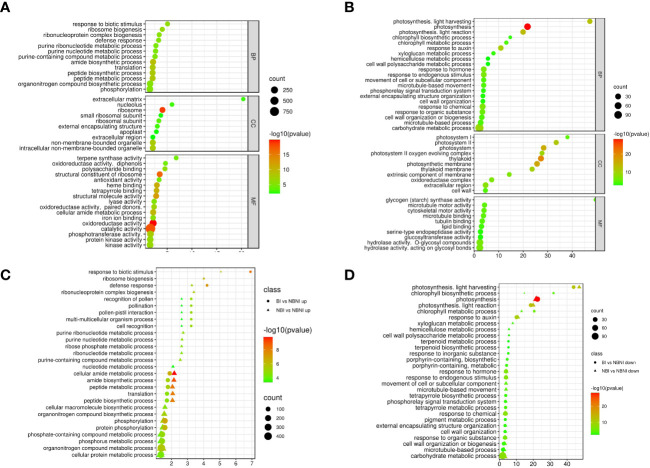
GO enrichment analysis for functional annotation of differentially expressed genes (DEGs). Pathway-enrichment bubble plots of upregulated **(A)** and downregulated **(B)** DEGs in *Fragaria vesca* Hawaii-4 leaves that were not treated with β-aminobutyric acid (BABA) and then infected with *Botrytis cinerea*. BP; biological process, MF; molecular function, CC; cellular compartment. Comparison of enriched GO-terms in the biological process category for NBI *vs.* NBNI (triangles) and BI *vs.* NBNI (circles) for upregulated **(C)** and downregulated **(D)** DEGs. Treatment combinations: *B. cinerea*-infected (NBI; No-BABA and Infected), mock-infected (NBNI; No-BABA and Non-Infected), BABA-treated and *B. cinerea* infected (BI; BABA and Infected), and BABA-treated and mock-infected (BNI; BABA and Non-Infected). X-axis shows log2-fold enrichment values. Plotted terms are selected based on log2 > 1 enrichment and P < 0.005. A comprehensive list of enriched terms can be found in **Supplementary Table S4**).

We identified the biological processes, cellular components, and molecular functions that were most strongly affected by *B. cinerea* infection in leaf tissue of plants that had not been treated with BABA. We did this by computing the enrichment of GO (Gene Ontology) terms of DEGs in infected and mock-infected plants (NBI *vs*. NBNI). Among upregulated genes (absolute log2-fold change values > 1) in the ‘biological process’ category we identified 124 significantly enriched GO categories (P-value < 0.05). The most enriched GO terms with the lowest P-values were ‘response to biological stimulus’, ‘ribosome biogenesis’ and ‘defense response’. (**Supplementary Table 4** and **Figure 4A**). Among the most enriched GO terms for downregulated genes (**Figure 4B**) in the ‘biological process’ category were ‘photosynthesis’, ‘photosynthesis, light harvesting’ and ‘response to auxin’. For molecular function, the most enriched GO terms for upregulated DEGs included ‘oxidoreductase activity’ and ‘terpene synthase activity’, while ‘hydrolase’ and ‘glycogen (starch) synthase activity’ were significantly downregulated DEGs. For cellular components, ‘extracellular matrix’ and ‘ribosome’ were among the most enriched upregulated categories, while ‘thylakoid’, ‘extracellular region’ and ‘photosystem’ were among the most enriched downregulated DEGs.

To identify any differences in GO-enrichment for BABA-treated plants compared to non-treated plants, we ranked enriched GO terms for biological process for the NBI *vs*. NBNI and BI *vs*. NBNI comparisons of up- and downregulated genes (**Supplementary Table 5**) according to log2-values (odds ratio) in the same enrichment bubble plot (**Figures 4C, D**). Most GO terms showed similar enrichment in infected plants (both BI and NBI) relative to non-infected plants not treated with BABA (NBNI). This was true for both up- and downregulated DEGs. However, a few terms showed different enrichment: for BI relative to NBI, terms like ‘defense response’, and ‘response to biologic stimulus’ were enriched in upregulated DEGs, while for NBI ‘purine ribonucleotide biosynthetic process’ and ‘ribosome biogenesis’ were enriched. For downregulated DEGs ‘chlorophyll biosynthetic and metabolic process’ was most enriched for BI, while ‘xyloglucan metabolic process’ was most enriched for NBI (**Figures 4C, D**). For the direct BI *vs.* NBI comparison, there was a significant enrichment of genes involved in photosynthesis, suggesting that BABA strenghtened the *B. cinerea* -mediated downregulation of this biological process.

**Figure 5:**
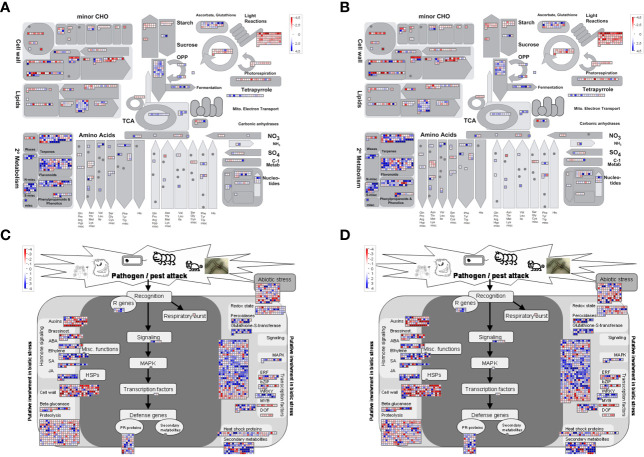
Visualization of enriched metabolic pathways in *Fragaria vesca* leaves after β-aminobutyric acid (BABA) treatment and *Botrytis cinerea* infection. Differentially expressed genes (DEGs) were displayed onto metabolic pathways using the MAPMAN software (**A, B**: central metabolism pathways; **C, D**: biotic stress pathways). **(A, C)** DEGs in *Botrytis cinerea*-infected *vs*. mock-infected plants [No-BABA and infected (NBI) *vs*. No-BABA and non-infected (NBNI)]. **(B, D)** DEGs in BABA-treated and *Botrytis cinerea*-infected plants *vs*. mock-infected plants (BABA and infected (BI) *vs*. NBNI). Blue cells: upregulation compared to NBNI; red cells: downregulation compared to NBNI.

We then did a Mapman analysis for a more detailed functional categorization of DEGs and visualization of associated metabolic pathways. This analysis confirmed the downregulation of photosynthesis-related genes (**Supplementary Figure S7**), carbohydrate metabolism, and cell wall-related genes in infected *vs*. uninfected plants not treated with BABA (NBI *vs*. NBNI), while genes involved in secondary metabolism, like phenolics and phenylpropanoid pathway, flavonoids and terpenoids, were upregulated (**Figure 5A** and **Supplementary Figures S5**, **S6**). Visualization of DEGs involved in the biotic stress pathway showed that many bins involved in defense signaling were differently regulated, and that genes involved in auxin signaling were downregulated while JA-signaling were upregulated (**Figure 5C**). Genes involved in defense, like R-proteins and PR-proteins and WRKY transcription factors, and secondary metabolites were generally upregulated, while genes involved in proteolysis and cell wall organization were downregulated. Like the GO enrichment analysis, the Mapman and KEGG analysis (**Supplementary Figure S9**) showed that the same pathways were induced in all infected plants, irrespective of BABA treatment, although individual bins within each pathway were differently expressed (**Figures 5B, D** and **Supplementary Figures S5–S7**).

## Discussion

Susceptibility of *F. vesca* to *B. cinerea* infection depends on genotype, ontogenic stage, tissue type, fruit ripening stage, and leaf age. Analogous to white berries and open flowers (Haile et al., 2019), leaves have a relatively slow disease progression with symptoms appearing 4-5 days after *B. cinerea* infection. In this study we characterized systemic effects of the well-characterized defense elicitor BABA applied as soil drench, by analyzing *B. cinerea* infection and molecular response in leaves of *F. vesca* plants. Surprisingly, we found that BABA induced increased susceptibility to this pathogen.

### BABA induces systemic susceptibility in strawberry

When applied as a soil drench BABA has previously been reported to induce systemic resistance against *B. cinerea* in distal organs like leaves and fruits in tomato (Luna et al., 2016; Wilkinson et al., 2018), and against bacteria in leaves of Arabidopsis and common bean (Po-Wen et al., 2013; Martínez-Aguilar et al., 2016). However, one previous report has shown that BABA seed treatment increased susceptibility to *B. cinerea* in leaves of tomato (Worrall et al., 2012). Still, it was a surprise to us that BABA soil drench induced systemic susceptibility in *F. vesca* leaves. As reported for several other crops, we observed that *F. vesca* plants soil drenched with BABA showed significantly reduced plant growth compared to control plants. When applied as a foliar spray BABA did not induce susceptibility but rather moderate (but non-significant) local resistance in *F. vesca* leaves, and plant growth was not affected. Because the BABA concentrations used in our foliar applications had no direct antifungal effect (**Supplementary Figure S3**), the reduced colonization by *B. cinerea* in these experiments was probably due to locally induced defense responses. Although most previous studies have shown a positive effect of BABA on plant resistance, the systemic susceptibility versus local resistance effect we observed is in line with some previous studies showing that the effect of BABA may depend on the application method, the developmental stage of the plant, and on plant genotype (Sharma et al., 2010; Alexandersson et al., 2016; Wilkinson et al., 2018; Yassin et al., 2021).

### No large transcriptional differences between untreated and BABA-treated plants after infection

RNA-Seq showed that *B. cinerea* induced similar transcriptional reprogramming in leaves of untreated *F*. *vesca* plants (NBI *vs*. BNI) and BABA-treated plants (BI *vs.* NBNI). The GO enrichment analysis of all DEGs showed that all the major pathways were similarly enriched between BABA-treated and untreated plants (**Figure 4**) after infection, although a low number of genes involved in ‘response to biologic stimulus’ were significantly upregulated in BABA-treated plants, while genes involved in ‘cell wall polysaccharide metabolic process’, ‘hemicellulose metabolic process’ and ‘xyloglucan metabolic process’ were significantly downregulated in untreated plants. Genes involved in photosynthesis and primary carbohydrate metabolism were downregulated in F. vesca leaves upon B. cinerea infection. Similar responses to B. cinerea infection are seen in Arabidopsis, grape berries, lettuce, and cucumber (Windram et al., 2012; De Cremer et al., 2013; Agudelo- Romero et al., 2015; Kong et al., 2015). Thus, it appears that plants infected by B. cinerea shut down carbohydrate metabolism, possibly to reduce the availability of easily metabolized carbohydrates to the pathogen (Göhre et al., 2012). For downregulated genes, BABA-treated plants showed enrichment for GO-terms like ‘chlorophyll biosynthetic’ and ‘chlorophyll metabolic’ process compared to non-treated plants, and for the direct comparison of DEGs between BI and NBI the term ‘photosynthesis’ was highly enriched, suggesting photosynthesis is more downregulated in BABA-treated plants. Photosynthesis play an important role in resistance to disease and can be another explanation for the increased susceptibility we observed. For example, genes related to photosynthesis were maintained at a relatively stable level in leaves of a resistant rice cultivar compared to leaves of a susceptible cultivar in the early stage of Rhizoctonia solani infection (Zhang et al., 2017). Interestingly, a recent study concluded that in addition to leaf age and low hydrogen peroxide levels, high chlorophyll and carotenoids levels were the best indicators for leaf resistance to B. cinerea in strawberry leaves (Meng et al., 2019). The Mapman analysis confirmed the GO enrichment data, showing that major metabolic pathways were similarly induced following infection in both BABA-treated and untreated plants, although some bins within each pathway were differently expressed between treatments. BABA has been shown to induce expression of genes involved in phenylpropanoid and salicylic acid (SA) biosynthesis associated with reduced decay incidence when applied locally (Li et al., 2021). However, except for the reduced expression of genes involved in photosynthesis the induced susceptibility observed in our study does not seem to be explained by induction or repression of major metabolic pathways, but rather by differential expression of individual genes that might induce susceptibility. For example, our Venn-analysis identified 229 upregulated and 445 downregulated genes that were unique for BABA-treated and infected plants (BI) *vs*. mock-infected plants with or without BABA treatment (NBNI and BNI). Some of the downregulated genes encoded proteins involved in isoprenoid metabolic and catabolic processes that could partly explain the increased susceptibility compared to untreated plants, as isoprenoids are secondary metabolites with a role in defense.

Also, individual genes that might induce susceptibility were differently expressed: our BABA-treated plants showed induction of WRKY40-like transcription factors that are similar to the Arabidopsis WRKY40 gene which results in increased susceptibility to *B. cinerea* when overexpressed with WRKY18 and WRKY60 (Xu et al., 2006).

### Major defense pathways are induced by *Botrytis cinerea* infection in leaves

In contrast to the unexpected systemic susceptibility induced by BABA soil drench, *B. cinerea* induced well-known transcriptional defense responses in leaves of both BABA-treated and untreated plants. These responses included changes in genes involved in pathogen recognition, cell-wall modification, defense signaling, biosynthesis and metabolism of jasmonic acid (JA), ethylene (ET) and auxin, and secondary metabolite pathways (**Figures 4**, **5** and **Supplementary Table 4**). The JA- and ET-pathway genes are known to be involved in positive regulation of defense against *B. cinerea* and were also induced in infected leaves in our study. In contrast, auxin-responsive genes were downregulated, as was also seen in resistant white berries (Haile et al., 2019).

Pattern recognition receptors (PRR) are receptor-like kinases (RLKs) that are localized in the plasma membrane and involved in the first line of defense against pathogens. We found that several of the RLK-subclasses previously reported to be involved in pathogen perception also were induced in *F. vesca* leaves upon *B. cinerea* infection. Wall-associated receptor kinases (WAKs) are involved in pathogen recognition, cell wall integrity, and defense against plant diseases (Kohorn, 2015; Bacete et al., 2017). For example, silencing of WAK4 in *Rosa chinensis* reduces pathogen resistance to *B. cinerea* (Liu et al., 2021). In *F. vesca* leaves, we found that 11 out of 40 wall-associated kinase/wall-associated kinase-like (WAK/WAKL) transcripts were upregulated upon *B. cinerea* infection, suggesting they have a role in defense. WAKs have also been shown to be upregulated by *B. cinerea* in berries (Haile et al., 2019), but these are not identical to the WAKs we detected in leaves, suggesting tissue-specific regulation of WAKs in *F. vesca*. Leucine-rich repeats (LRR)-RLKs have been shown to perceive DAMPs that are released after cell wall damage and are thus thought to be involved in cell wall-mediated immune responses. We found induction of 18 probable LRR receptor-like serine/threonine-protein kinases and 11 L-type lectin-domain-containing receptor kinases (LecRKs) in *F. vesca* leaves. Like in berries, several of the MAPK-kinases (20 out of 61) involved in intracellular immune signaling following pathogen infection were induced.

### Transcription factors induced by *Botrytis cinerea* in leaves

Transcription factors (TFs) play an important role in defense against pathogens by regulating expression of genes involved in stress responses, such as cell wall modulation, pathogen perception and response, hormone pathways, and production of secondary metabolites. The most highly upregulated TF-classes in *F. vesca* leaves were the ethylene-responsive (ERFs), WRKY-class, bHLH, and MYB TFs (**Figures 5C, D** and **Supplementary Table 2**). In Arabidopsis, negative regulation of ABA signaling by WRKY33 and positive regulation of the jasmonate-mediated signaling pathway are critical for defense against *B. cinerea* (Liu et al., 2015; Chen et al., 2021). In our study, WRKY75 was significantly upregulated in leaves infected with *B. cinerea* and may thus play a similar role in *F. vesca* as in Arabidopsis. However, the WRKYs we identified in *F. vesca* leaves were not identical to those previously identified in white berries (Haile et al., 2019), again suggesting tissue-specific responses to infection in *F. vesca*.

### Cell wall modification by *Botrytis cinerea*

Regulation of cell wall assembly and disassembly is important for resistance against *B. cinerea* in tomato and strawberry berries (Cantu et al., 2007; Haile et al., 2019). Many genes involved in cell wall metabolism were differently expressed in *F. vesca* leaves upon *B. cinerea* infection, suggesting that infection induces structural rearrangements of the cell wall. For example, five probable xyloglucan endotransglucosylases (XTHs), which are involved in cell wall modulation and repair, were induced in response to infection, while seven were downregulated. Several studies have demonstrated fungus-mediated transcriptional shut-down of these enzymes, probably to facilitate fungal colonization (Miedes et al., 2014), while in other studies, the GO term ‘xyloglucan metabolic process’ was enriched for genes induced by fungal infection of susceptible plants (Ksiażkiewicz et al., 2021). Interestingly, plants not treated with BABA were also more enriched for ‘xyloglucan metabolic process’ in downregulated genes than BABA-treated plants and this might partly explain the difference in susceptibility to *B. cinerea* between BABA-treated and untreated plants.

Microbial pathogens, including *B. cinerea*, secrete polygalacturonases (PGs) which depolymerize pectine polymers in the plant cell wall. In response, plants produce polygalacuronase-inhibiting proteins (PGIPs) that counteract the activity of polygalacturonases and confer resistance against *B. cinerea* infection in a number of species, including grape and tomato (Kalunke et al., 2015). In *Fragaria chilesensis*, upregulation of PGIP and the β-1,3-glucanase BG correlated with reduced incidence of postharvest grey mold after preharvest application of chitosan and MeJA (Saavedra et al., 2017). The only two PGIPs we detected in the leaf transcriptome were upregulated after infection, suggesting a similar response in *F. vesca* leaves. The level of methylesterification of pectins regulated by pectin methylesterases (PMEs) and pectin methylesterase-inhibiting proteins (PMEIs) affects susceptibility to pathogen attack (Lionetti et al., 2012; Bellincampi et al., 2014). PMEIs have been shown to reduce susceptibility to pathogens such as *B. cinerea* by inhibiting PMEs to increase pectin methylesterification (Lionetti et al., 2017). However, PMEs can also induce defense against pathogens by generating demethylesterified oligogalacturonides (OGAs) (Brutus et al., 2010). In *F. vesca*, ectopic expression of the *F. ananassa* pectin methylesterase PE1 partly demethylates OGAs that are perceived as DAMPs by WAKs and confer partial resistance against *B. cinerea* (Osorio et al., 2008; Osorio et al., 2011).

In our study both PMEs and PMEIs were highly differently regulated, suggesting strong regulation of pectin methylesterification upon *B. cinerea* infection in *F. vesca* leaves. Thus, transcripts controlling pectin methylesterification in *F. vesca* seem to be partly activated and partly deactivated following leaf infection by *B. cinerea*.

We found several expansins to be downregulated in *F. vesca* leaves after *B. cinerea* infection, although two expansin-like B1 transcripts were strongly induced. Most expansins are cell wall remodeling agents which enables cell expansion through non-enzymatically relaxation of the cell wall (S. AbuQamar, 2014; Marowa et al., 2016). The role of cell wall disassembly in ripening-associated susceptibility to *B. cinerea* infection was demonstrated in tomato, where suppression of the polygalacturonase LePG and the expansin LeExp1 in ripening fruits increased resistance to *B. cinerea* (Cantu et al., 2007).

*Fragaria vesca* leaves infected with *B. cinerea* also showed upregulation of most lignin-associated cinnamoyl-CoA reductase (CCR) or reductase like genes, which belong to the large group of oxidoreductases - one of the most enriched GO-terms in upregulated DEGs. Upregulation of these genes, that encode an enzyme in the monolignol pathway, suggests that cell-wall strengthening processes were activated in the leaf in response to infection. In a previous study, upregulation of CCR correlated with cell wall strengthening and production of the antimicrobial phytoalexin resveratrol and inhibition of *B cinerea* growth in unripe grapevine berries (Kelloniemi et al., 2015). The GO term ‘oxidoreductase’ includes ‘peroxidases’ which was also enriched upon *B. cinerea* infection in *F. vesca* leaves. Peroxidases fortify the cell wall by polymerizing monolignols into lignin.

### PR proteins are important for leaf defense

Pathogenesis-related (PR) proteins are primarily induced by pathogens and can be divided into 17 families based on their primary structure, function, and biological properties. Several main classes of PR proteins cause damage to fungal cell walls (Sels et al., 2008; AbuQamar et al., 2016; Zeilinger et al., 2016). Our Mapman-analysis showed a strong enrichment for PR-proteins among the upregulated genes, including most major allergen Pru ar 1-like genes, a type of PR-10 protein involved in response to biotic stimulus. These genes are more highly expressed in resistant, white *F. vesca* berries than in the more susceptible red berries (Haile et al., 2019). Furthermore, we found three Fra a 1 allergen of the PR-10 class to be highly upregulated in *F. vesca* leaves. Knock-down of these genes in *F. ananassa* reduces expression of FvPAL and FvCHS as well as metabolic intermediates of the flavonoid pathway, suggesting that these genes activate the flavonoid biosynthesis pathway which is involved in defense (Muñoz et al., 2010).

The GO term oxidoreductase was enriched after *B. cinerea* infection in leaves. Germin-like proteins (GLPs) are classified as PR proteins in the PR-16 family. GLPs can be induced by fungal pathogens and have oxidoreductase activity resulting in H_2_O_2_ production, induction of JA-mediated signaling, and activation of defense against pathogens (Pei et al., 2019). In contrast to what has previously been demonstrated in white berries (Haile et al., 2019), expression of five out of 14 GLPs were strongly induced in infected *F. vesca* leaves, suggesting that leaves have a stronger H_2_O_2_ defense response upon infection than berries.

### Genes involved in secondary metabolite production are induced by *Botrytis cinerea* in leaves

As in white berries, important steps in the phenylpropanoid pathways were upregulated in *F. vesca* leaves upon infection (**Supplementary Figure S5**). Plants activate the phenylpropanoid pathway when pathogens breach the cell wall, leading to the synthesis of precursors for many secondary metabolites important for defense. This includes signaling molecules, antimicrobial phenolic compounds like flavonoids and ellagitannins, and monolignol precursors for lignin biosynthesis (Yadav et al., 2020).

Phenolic compounds play a major role in resistance in immature berries of strawberry, and the levels of flavonoids like proanthocyanidins and flavan-3-ols diminish as the berries ripen, increasing their susceptibility to *B. cinerea* (Mikulic-Petkovsek et al., 2013; Nagpala et al., 2016). Two shikimate dehydrogenase genes were also induced by *B. cinerea* infection in *F. vesca* leaves in our study. These genes are involved in the production of ellagitannin, which is enriched in unmature berries and leaves after pathogen infection (Mamaní et al., 2012). In addition, genes involved in the biosynthesis of defense metabolites such as terpenoids were upregulated, reflected by the enrichment of the GO-term ‘terpene synthase’ in upregulated genes of non-BABA treated plants, suggesting that several defense systems were activated in *F. vesca* leaves upon *B. cinerea* infection. Terpenes are a diverse group of compounds including molecules with antimicrobial activity. The importance of terpene synthases in defense was recently demonstrated in berries of *F. ananassa*, where overexpression of the terpenoid synthase *FaTPS1* increased production of the sesquiterpene germacrene D, and improved resistance to *B. cinera* in strawberry fruits (Zhang et al., 2022).

## Conclusion

*Fragaria vesca* leaves showed similar transcriptional reprogramming following *B. cinerea* infection as previously seen in flowers and unmature berries of *F. vesca*, with induction of major metabolic pathways important for defense. However, we also found leaf-specific responses to *B. cinerea* infection that might reflect the higher resistance of leaves compared to mature berries. Furthermore, we show that, similar to berries in strawberry and grapes, BABA increased disease resistance when applied locally to leaves. However, in contrast to the Arabidopsis-*B. cinerea* pathosystem, BABA reduced plant growth and induced susceptibility to *B. cinerea* infection when applied systemically as a soil drench. This effect was associated with reduced expression of genes involved in photosynthesis compared to non-treated plants, suggesting that BABA enhanced the B. cinerea induced downregulation of this biological process. Thus, our study adds to the evidence that the effect of defense elicitors such as BABA on plant resistance varies among tissue types, development stages, application methods, and genotypes.

## Experimental procedures

### Plant growth

*Fragaria vesca* ‘Hawaii-4’ and *F. vesca* ‘Alexandria’ accessions were cultivated in a growth room with 14 h light (~100 μmol m^-2^ s^-1^ photosynthetically active radiation (PAR) at 24 °C) and 10 h darkness (19°C) at 40-45% relative humidity. We also used *Fragaria × ananassa* in one experiment to test if BABA soil drench affected resistance in commercial strawberry. Plants were grown in topsoil in 400 mL pots and were not subjected to any kind of chemical treatments (pesticides, fungicides, or fertilizers) that could interact with the plants’ phenotypic responses to BABA. For the infection experiments we used 8- to 12-week-old plants grown from seeds to ensure that the plants in each experimental batch were in the same developmental stage. Plants were about 10-12 cm tall (measured from the soil surface) at the start of the experiments, bearing 6-10 trifoliate leaves.

### Treatment with BABA

β-aminobutyric acid (BABA, Sigma-Aldrich, #A44207-5G) was dissolved in distilled H_2_O. For soil-drench treatments, 50 ml of BABA solution was added to each pot to a final concentration of 0 mM (control), 0.05 mM, 0.15 mM, 0.2 mM, 0.3 mM or 0.6 mM. Each pot contained one plant and received a single soil-drench treatment. For above-ground treatment, 0.3 mM BABA solution was sprayed onto leaves until run-off. BABA treatment was always performed two days before infection with *B. cinerea*.

### *Botrytis cinerea* inoculum preparation and infection

*Botrytis cinerea* isolate Bc101 was grown on potato dextrose agar plates in darkness. Spores on 4- to 5-week-old plates were harvested by pouring liquid potato dextrose broth (PDB) media onto the plates and collecting the liquid with a pipette. The spore suspension was passed through a sterile 70 µM nylon mesh and centrifuged at 3000g for 1 min, before the spore pellet was re-suspended in fresh PDB media. Spores were counted using a hemocytometer and the suspension was adjusted to a concentration of 10^6^ spores mL^-1^. The spore suspension was supplemented with 0.02% Tween-20 and sprayed on plants using a hand sprayer until run-off. PDB media supplemented with 0.02% Tween-20 was used for mock infections. Sprayed plants were placed in a plastic tray, covered with transparent polypropylene bags to maintain high humidity that promotes infection, and incubated in a plant growth room (14 h light and 10 h darkness at room temperature, ~100 μmol m^-2^ s^-1^ PAR) until symptoms appeared. Necrotic lesions on leaves usually began to appear 4-5 days after spore spraying. Student’s t-tests were used for statistical comparison of different treatment combinations for all phenotypic data.

### Isolation of RNA and DNA

About 100 mg of leaf tissue harvested 5 days post infection was ground in liquid N_2_ and used for DNA extraction using the DNeasy Plant Kit (#69106) in a QIAcube automated sample prep system according to the manufacturer’s instructions. Total RNA was isolated from 100 mg leaf tissue harvested 24 hours post infection using Spectrum™ Plant Total RNA Kit with minor modifications (Badmi et al., 2018). Briefly, 100 mg of tissue powder was mixed with preheated lysis buffer containing CTAB (2%), PVPP (2%), Tris-Cl (pH 8.0, 100 mM), EDTA (pH 8.0, 25 mM), NaCl (1 M) and β-mercaptoethanol (1%). The mixture was incubated at 65 °C for 8 min, with vortexing for the first 60 seconds. The lysate was then centrifuged for 10 min at 13,000 rpm, the supernatant was mixed with an equal volume of chloroform:isoamyl alcohol (24:1) and centrifuged again for 10 min at 4 °C. The supernatant was transferred to the kit’s filtration column (blue retainer ring), and from this step we followed the manufacturer’s instructions. On-column DNase I treatment was done to ensure DNA-free total RNA. Total RNA (500 ng) was used to prepare cDNA using iScript cDNA Synthesis Kit (#170-8891). SsoAdvanced Universal SYBR Green Supermix (#1725271) was mixed with genomic DNA or cDNA to perform RT-qPCR on the Applied Biosystems ViiA 7 system and determine the C_t_ values for each primer pair.

### RNA-seq analysis

*Fragaria vesca* Hawaii-4 plants were either drenched with 50 ml water or soil-drenched with 50 ml 0.2 mM BABA. After 48 hours, five plants of each treatment (water or BABA) were either mock-infected or infected with *B. cinerea* spores as described above, and leaves were harvested for RNA isolation 24 h later. Each plant served as a single biological replicate (n = 5). Isolated total RNA (0.3 µg) was used to prepare samples using TruSeq Stranded mRNA (Illumina, San Diego, CA, USA) according to the manufacturer’s instructions. Library preparation and cDNA sequencing was performed at the Norwegian Sequencing Centre (Oslo, Norway) on the Illumina NextSeq 500 system with 75 bp single end reads. Demultiplexed reads were trimmed and filtered using trimmomatic (version 0.38) with recommended settings (Bolger et al., 2014). Trimmed and filtered reads were aligned to the chromosome-continuity version 4.0.a1 of the *F. vesca* transcriptome (rosaceae.org) using hisat2 (version 2.1.0) (Putri et al., 2022). Gene count data were extracted using the htseq-count function from the HTSeq package (version 0.11.2) (Anders et al., 2015). Differentially expressed genes were identified using the R package EdgeR (version 3.32.1) and filtered using false discovery rate (alpha) < 0.05 and absolute log2-fold change values > 1 (Varet et al., 2016). Normalization was performed using the ‘trimmed mean of M values’ (TMM) method and p-values were adjusted using the Benjamini-Hochberg method (Benjamini, 2014). Differential expressed genes are visualized as volcano plot (adjusted p-values versus log2 fold-change based on edgeR data) (**Supplementary Figure S8**).

### QPCR DNA-based *Botrytis cinerea* quantification

Quantification of *B. cinerea* was performed using genomic DNA isolated from infected *F. vesca* leaves 5 days after infection, when symptoms started to appear. C_t_ values from *B. cinerea*-specific genomic DNA primers (Bc3F and Bc3R, **Supplementary Table S6**) were normalized against *F. vesca*-specific *EF1α* primers to obtain the relative *B. cinerea* levels in the tissue. For RNA-seq validation using qPCR, *EF1α* was used as the housekeeping control gene and relative expression levels of genes were determined using the ΔC_t_ method (Pfaffl, 2001) using primers in **Supplementary Table S6**.

### Functional categorization of DEGs using GO and Mapman enrichment analysis

Differential expressed genes with False discovery rate (FDR) FDR < 0.05 were analyzed for enriched GO-terms based on *Fragaria vesca* Whole Genome v4.0.a1 Assembly & Annotation (www.rosaceae.org) and Blast2GO. GO pathway enrichment plot was plotted with SRplot (www.bioinformatics.com.cn/en). The Mercator4 online tool (Schwacke et al., 2019) was used to functionally annotate and classify all the *F. vesca* transcripts into hierarchically structured bins, and combined with DEG analysis and displayed onto metabolic pathways with the MAPMAN software (Thimm et al., 2004). DEGs with FDR < 0.05 were plotted in Venn diagrams using InteractiVenn (Heberle et al., 2015).

## Data availability statement

The datasets presented in this study can be found in online repositories. The names of the repository/repositories and accession number(s) can be found below: https://www.ncbi.nlm.nih.gov/, PRJNA818508.

## Author contributions

RB, MB, and TTh designed the experiments. CF contributed with design of some experiments. RB cultivated the plants and performed the experiments. LH did infection assays on *F. ananassa* and helped with experiments. AE isolated DNA from leaf tissues and cultivated plants, YZ helped with performing the experiments, TTe did most of the bioinformatical work and TTe, TTh, and RB analyzed the RNA-seq data. TH provided plants and greenhouse space. RB wrote the first draft of the manuscript. PK critically reviewed and contributed to the writing of the manuscript. TTh wrote the final manuscript. All authors contributed to the article and approved the submitted version.
