# Supplementary material for: Transcriptional profiling of defense responses to Botrytis cinerea infection in leaves of Fragaria vesca plants soil-drenched with β-aminobutyric acid
Source: Front Plant Sci. 2022 Dec 8;13:1025422. doi: 10.3389/fpls.2022.1025422 (PMC9772985; doi:10.3389/fpls.2022.1025422)
Supplement: Supplementary file 1 [file DataSheet_1.pdf]

Supplementary Material

Supplementary Figures and Tables

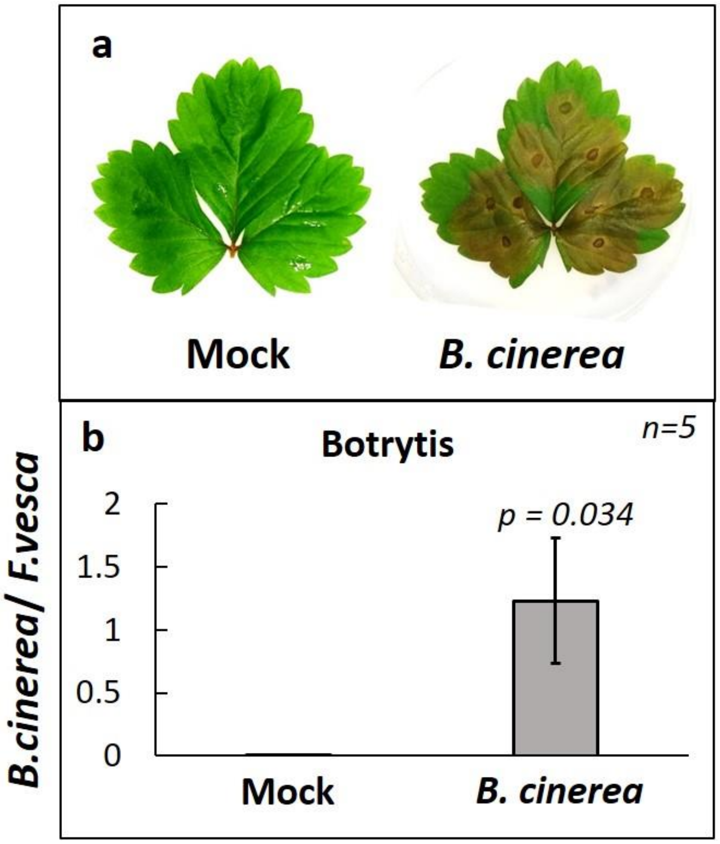

Supplementary figure S1: Quantification method for *Botrytis cinerea* infection and verification of pathogenicity in *Fragaria vesca* leaves. (a) Representative *F. vesca* 'Hawaii-4' leaves showing infection by *B. cinerea* 5 days after drop inoculation of fungal spores (right) or mock suspension (left). (b) Genomic DNA-based qPCR quantification of *B. cinerea* DNA relative to *F. vesca* DNA using genome-specific primers for *B. cinerea* (Bc3F and Bc3R) and *F. vesca* (EF1αF and EF1αR) in non-infected (Mock) and infected *(B. cinerea)* leaf samples. Y-axis values represents relative amount of *B. cinerea* DNA to *F. vesca* DNA. '*n'* = number of replicates (i.e. individual plants). Error bars show standard error (±1 SE). P-values report comparisons between infected and uninfected plants using Student's t-test.

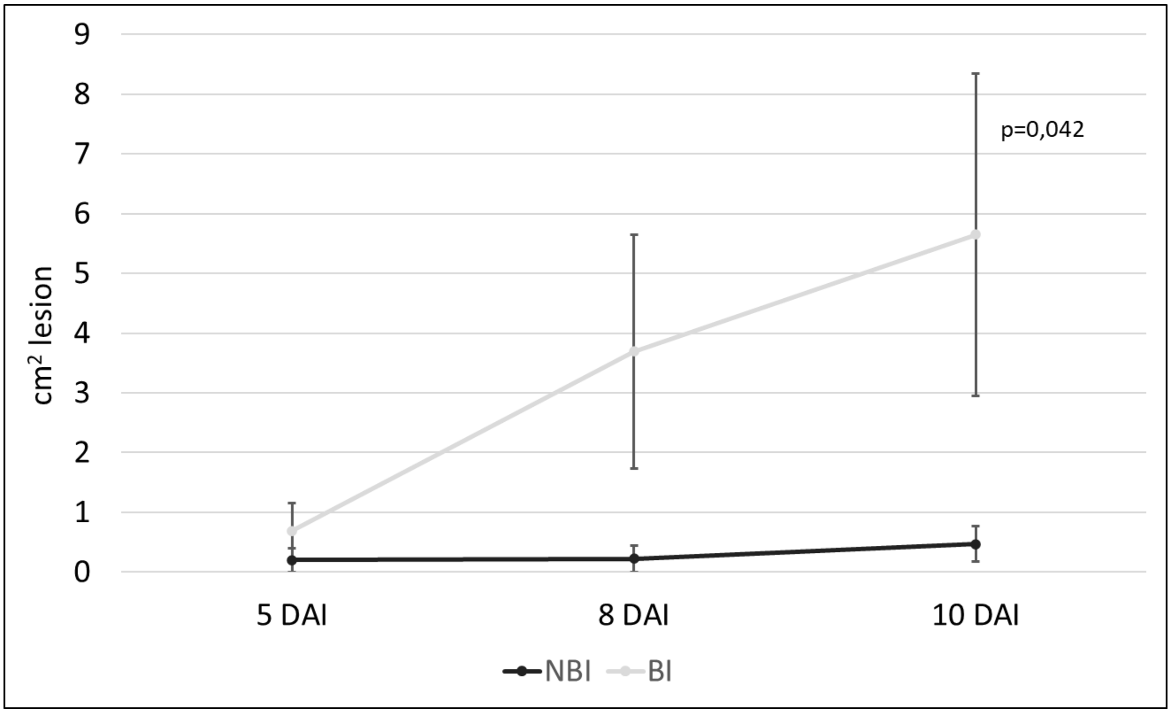

Supplementary figure S2: Effects of BABA soil drench on resistance of *Fragaria ananassa* to infection with *Botrytis cinerea*. Plants were soil drenched with water or 0.2 mM BABA 8 days prior to drop-inoculation of leaves with *B. cinerea* (No BABA and Infected (NBI) and BABA and Infected (BI), respectively). Measurement of lesion area (in cm^2) was done using ImageJ of images taken 5, 8, and 10 days after inoculation. Values show average lesion area on infected detached leaves from 16 individual plants (n = 16). Error bars show standard error (±1 SE). P-values report significant comparisons between control plants (NBI) and plants treated with BABA (BI) using Student's t-test.

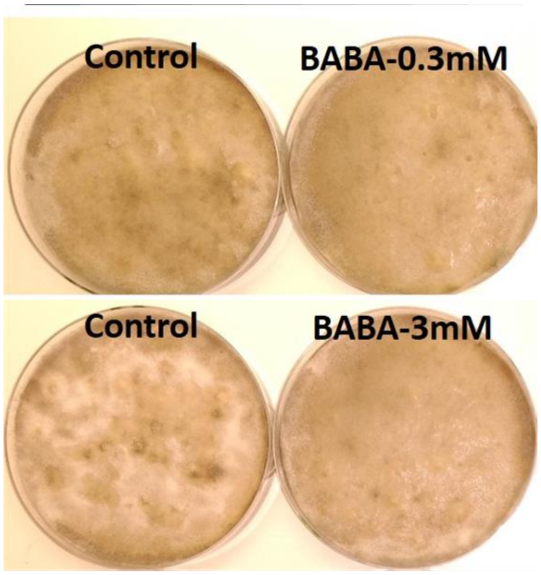

Supplementary figure S3: Direct effects of BABA on *Botrytis cinerea* growth. Growth of *B. cinerea* (brown mycelium) on potato dextrose agar amended with 0 mM, 0.3 mM, and 3 mM β-aminobutyric acid (BABA). A spore suspension of *B. cinerea* was spread on the agar and the plates were incubated in darkness for 4 weeks at room temperature.

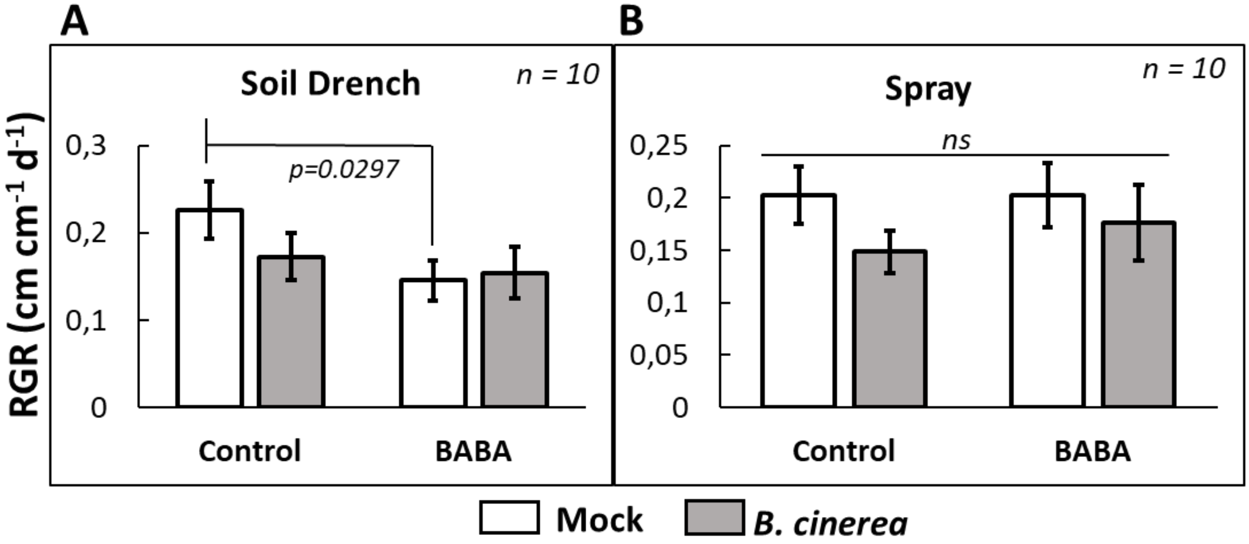

Supplementary figure S4: Effect of BABA on relative growth rate (RGR) of *Fragaria vesca* 'Hawaii-4' plants. RGR of plants that were (A) soil-drenched with 0.2 mM or (b) spray-treated with 0.3 mM β-aminobutyric acid (BABA). Control plants were treated with a suspension without BABA. All plants were subsequently infected with *Botrytis cinerea* spores or mock-infected. '*n'* = number of replicates (i.e. individual plants). Error bars show standard error (±1 SE). P-values report comparisons between treatment combinations using Student's t-test. '*ns*' = not statistically significant.

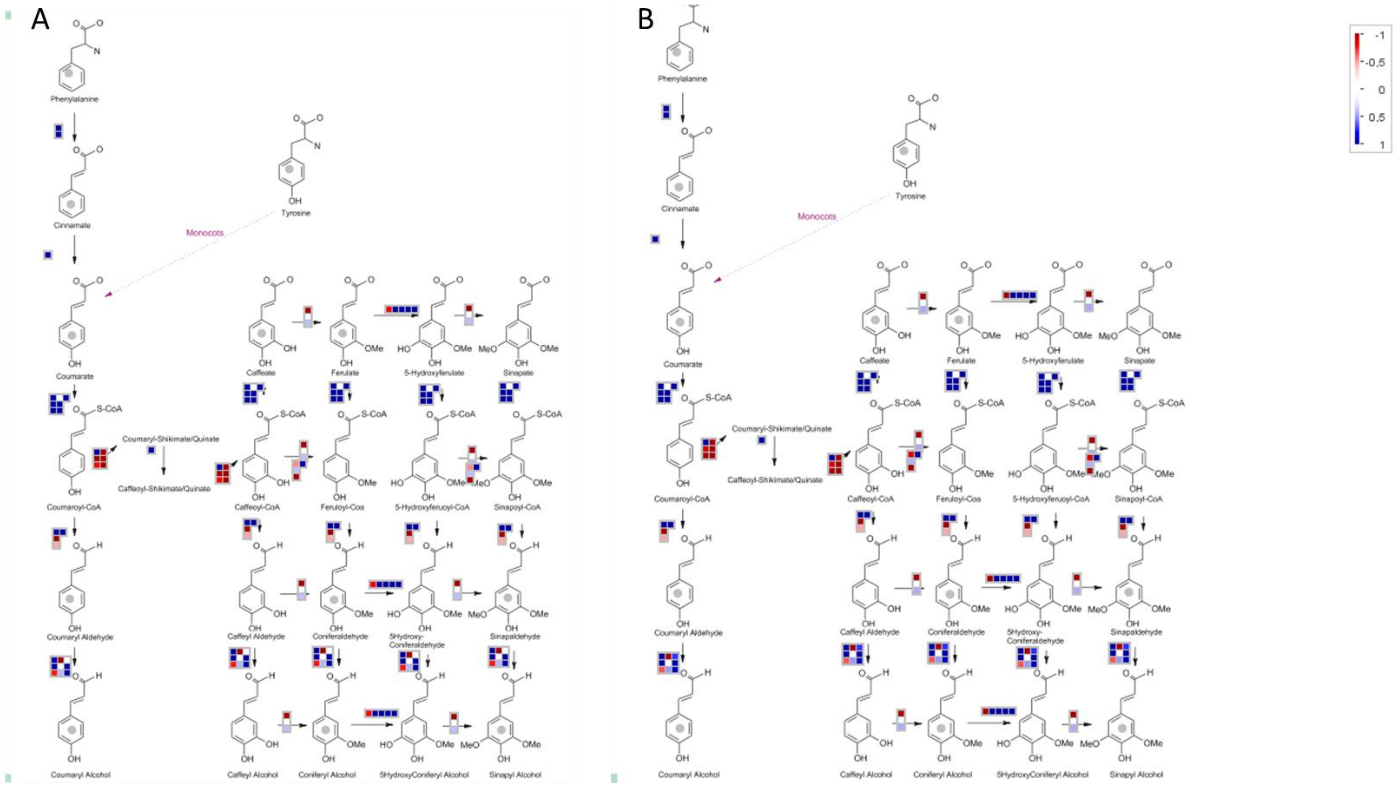

Supplementary figure S5. Visualization of DEGs in the phenylpropanoid pathway in *Fragaria vesca* leaves after β-aminobutyric acid (BABA) treatment and *Botrytis cinerea* infection. Differentially expressed genes (DEGs) were displayed onto metabolic pathways using the MAPMAN software. (A) *B. cinerea*-infected vs. mock-infected plants (No BABA and infected (NBI) vs. No BABA and non-infected (NBNI)). (B) BABA-treated and *B. cinerea*-infected plants vs. mock-infected plants (BABA and infected (BI) vs. NBNI). Blue cells: upregulation compared to NBNI; red cells: downregulation compared to NBNI.

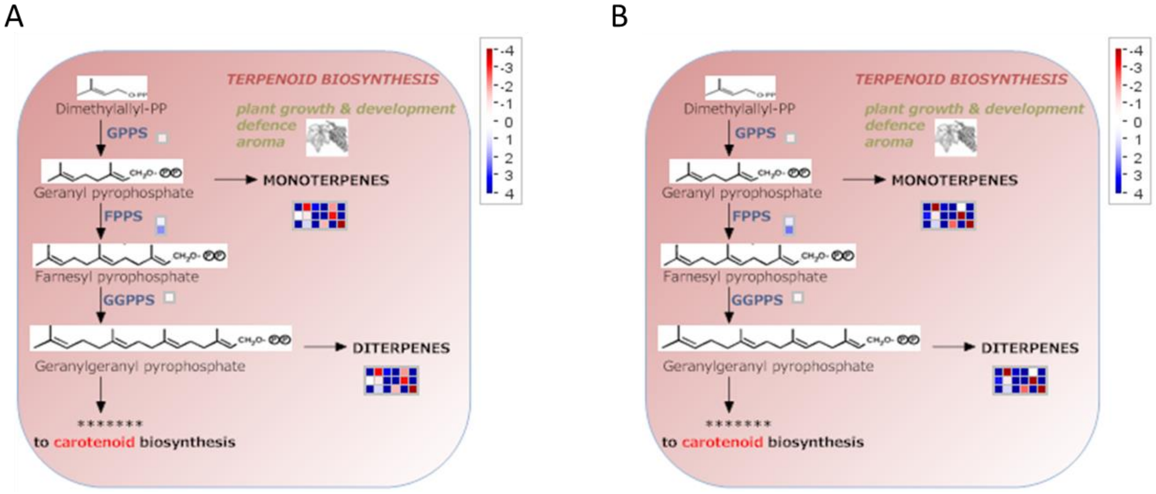

Supplementary figure S6. Visualization of DEGs in the terpenoid biosynthesis pathway in *Fragaria vesca* leaves after β-aminobutyric acid (BABA) treatment and *Botrytis cinerea* infection. Differentially expressed genes (DEGs) were displayed onto metabolic pathways using the MAPMAN software. (A) *B. cinerea*-infected vs. mock-infected plants (No BABA and infected (NBI) vs. No BABA and non-infected (NBNI)). (B) BABA-treated and *B. cinerea*-infected plants vs. mock-infected plants (BABA and infected (BI) vs. NBNI). Blue cells: upregulation compared to NBNI; red cells: downregulation compared to NBNI.

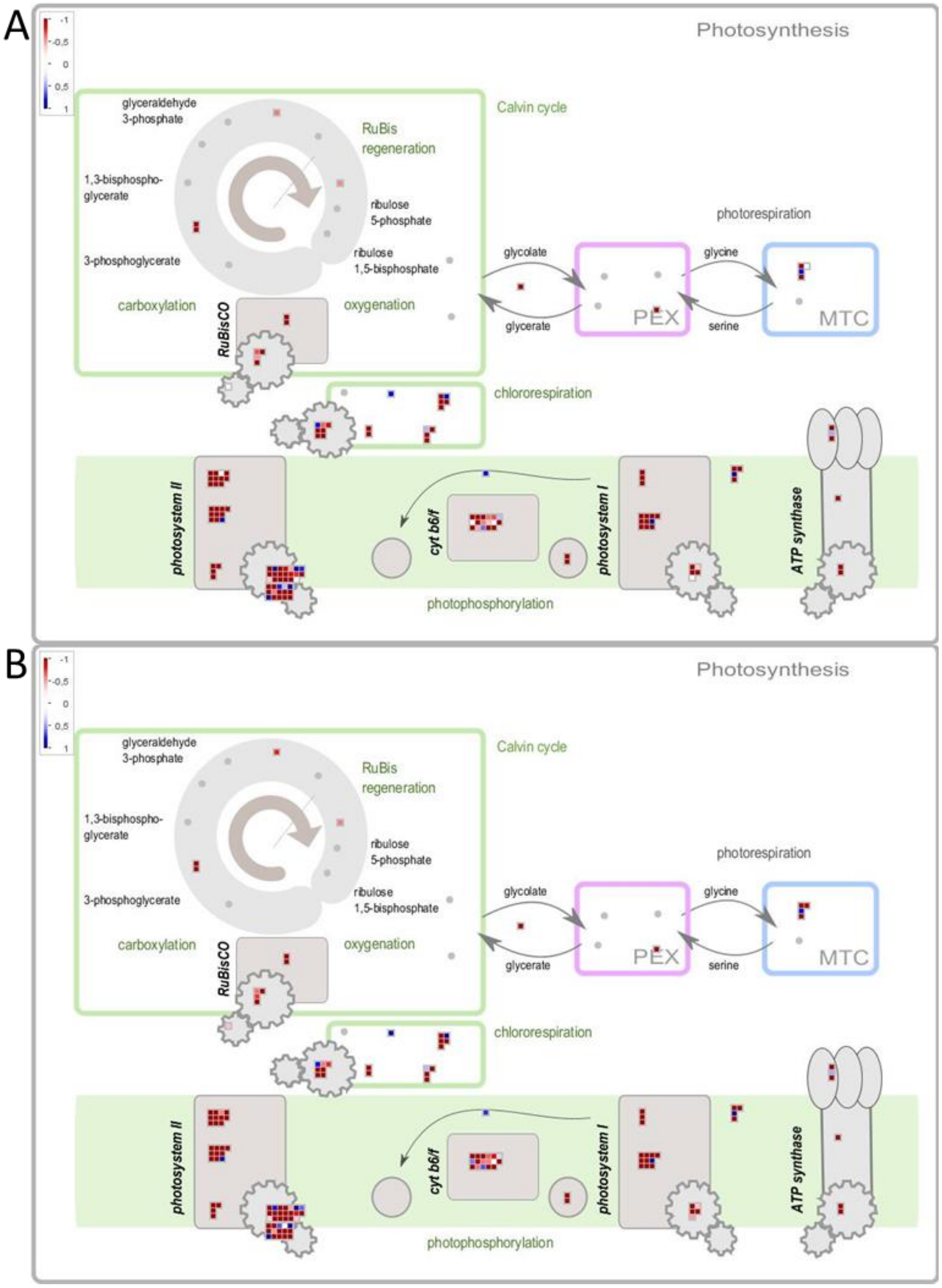

Supplementary figure S7. Visualization of enriched DEGs in photosynthesis in *Fragaria vesca* leaves after β-aminobutyric acid (BABA) treatment and *Botrytis cinerea* infection. Differentially expressed genes (DEGs) were displayed onto metabolic pathways using the MAPMAN software. (A) *B. cinerea*-infected vs. mock-infected plants (No BABA and infected (NBI) vs. No BABA and non-infected (NBNI)). (B) BABA-treated and *B. cinerea*-infected plants vs. mock-infected plants (BABA and infected (BI) vs. NBNI). Blue cells: upregulation compared to NBNI; red cells: downregulation compared to NBNI.

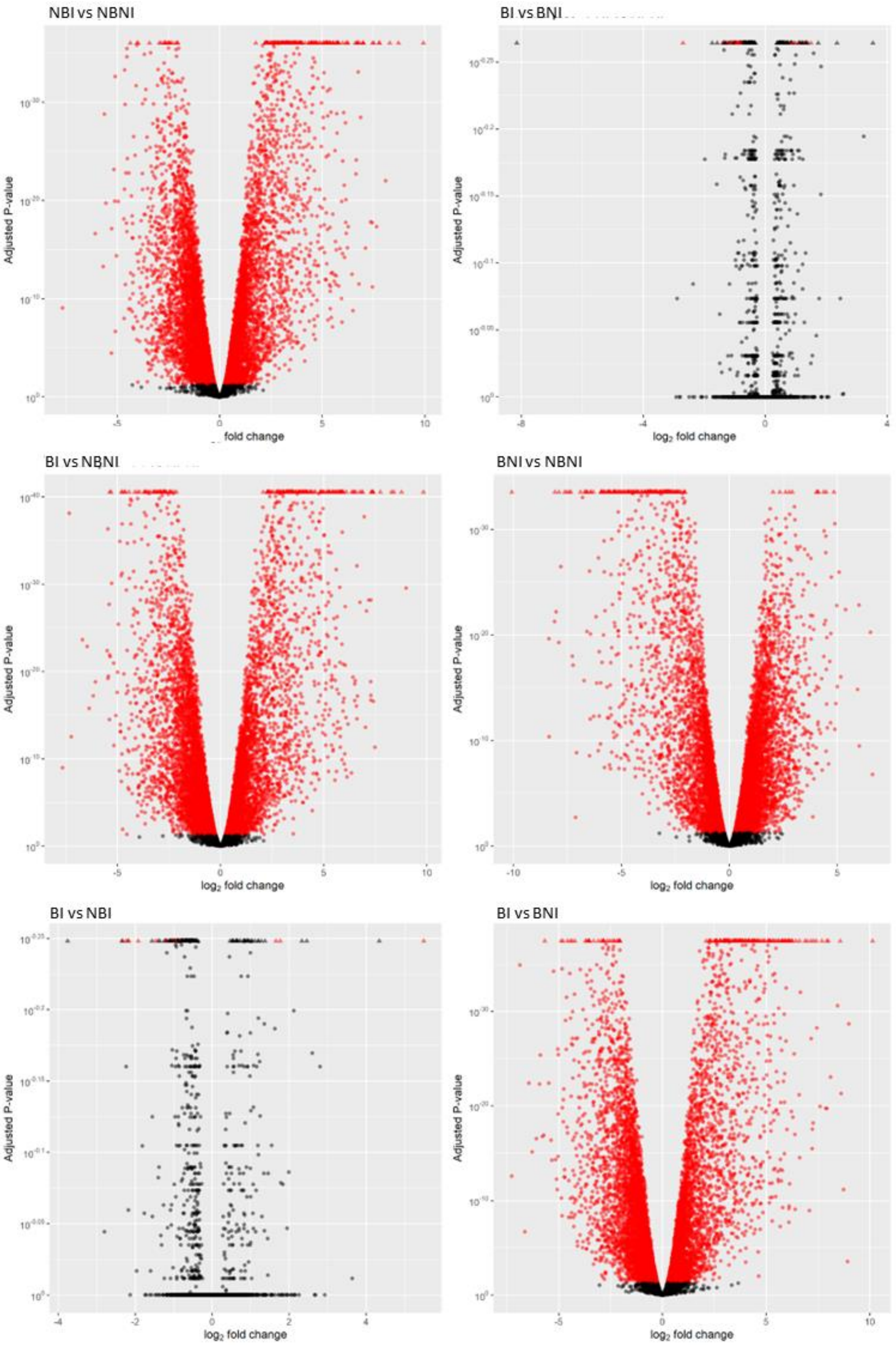
NBI vs NBNI
BI vs BNI
BI vs NBNI
BNI vs NBNI
BI vs NBI
BI vs BNI
Adjusted P-value
log2 fold change

Supplementary figure S8. Volcano plot (adjusted p-values versus log2 fold-change based on edgeR data) of transcripts in leaves of *Fragaria vesca* Hawaii-4 plants following BABA treatment and infection with *Botrytis cinerea*. Plants were treated with different combinations of 0.2 mM β-aminobutyric acid (BABA) as a soil drench and spray-inoculation of leaves with *B. cinerea* two days later. Transcripts with p-values < 0.05 are shown in red. (A) *Botrytis cinerea*-infected plants (No BABA and infected; NBI) vs. mock-infected plants (No BABA and non-infected; NBNI). (B) BABA-treated and mock-infected plants (BABA and non-infected; BNI) vs. NBNI. (C) BABA-treated and infected plants (BABA and infected; BI) vs. NBNI. (D) BNI vs. NBI. (E) BI vs. NBI. (F) BI vs. BNI.

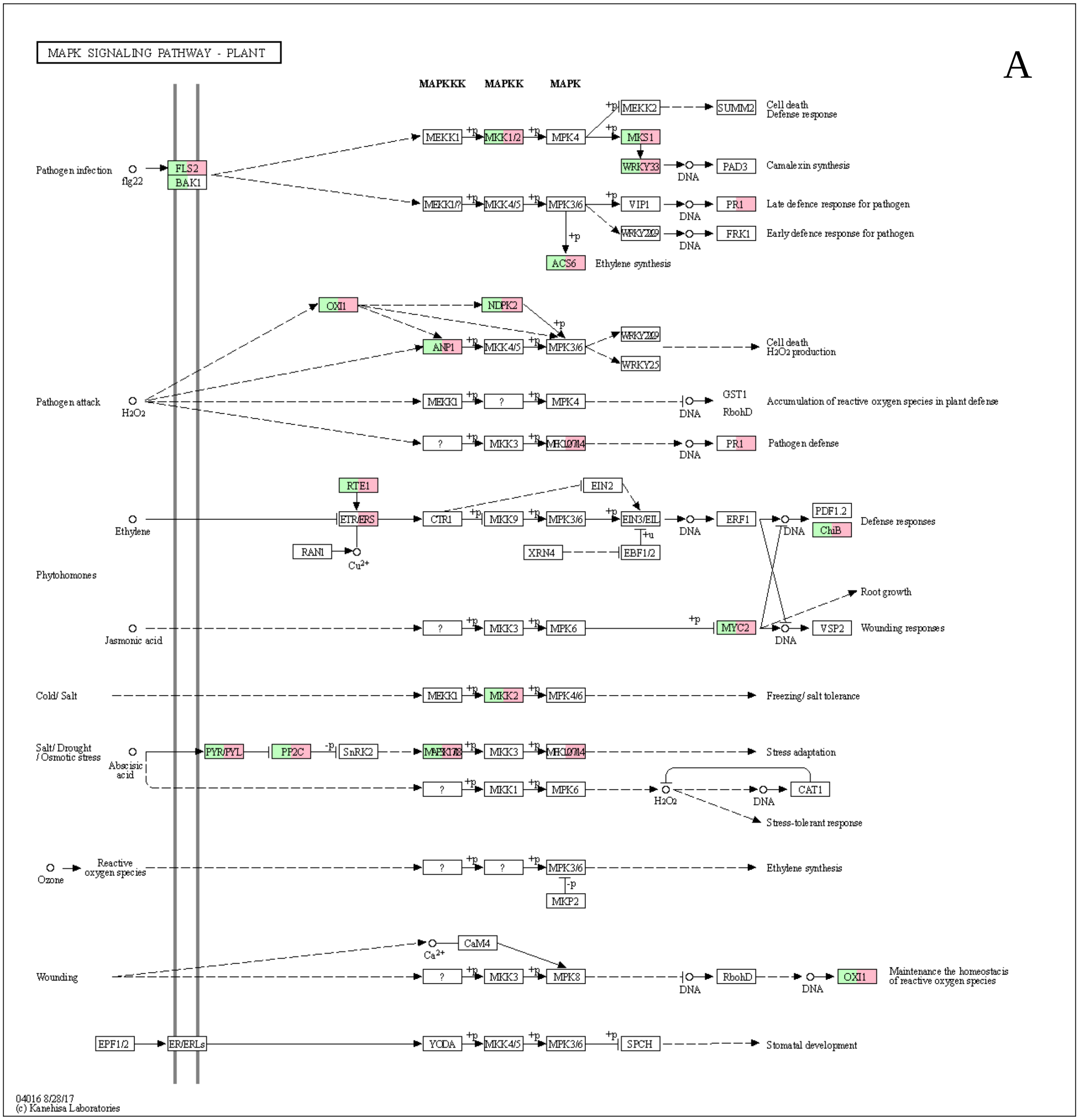
MAPK SIGNALING PATHWAY - PLANT
A
MAPKKK
MAPKK
MAPK
Pathogen infection
flg22
FLS2
BAK1
MEKK1
MKK1/2
MPK4
MEKK2
SUMM2
Cell death
Defense response
MKS1
WRKY33
DNA
PAD3
Camalexin synthesis
MKK4/5
MPK3/6
VIP1
PR1
Late defence response for pathogen
FRK1
Early defence response for pathogen
ACS6
Ethylene synthesis
OXI1
NDPK2
ANP1
WRKY25
H2O2 production
Pathogen attack
GST1
RbohD
Accumulation of reactive oxygen species in plant defense
MKK3
Pathogen defense
RTE1
EIN2
Ethylene
ETR/ERS
CTR1
MKK9
EIN3/EIL
ERF1
PDF1.2
ChiB
Defense responses
RAN1
XRN4
EBF1/2
Phytohomones
Root growth
Jasmonic acid
MPK6
MYC2
VSP2
Wounding responses
Cold/ Salt
MKK2
MPK4/6
Freezing/ salt tolerance
Salt/ Drought / Osmotic stress
Abscisic acid
PYR/PYL
PP2C
SnRK2
MKK1
CAT1
Stress adaptation
Stress-tolerant response
Ozone
Reactive oxygen species
MKP2
Wounding
CaM4
MPK8
Maintenance the homeostacis of reactive oxygen species
EPF1/2
ER/ERLs
YODA
SPCH
Stomatal development
04016 8/28/17
(c) Kanehisa Laboratories

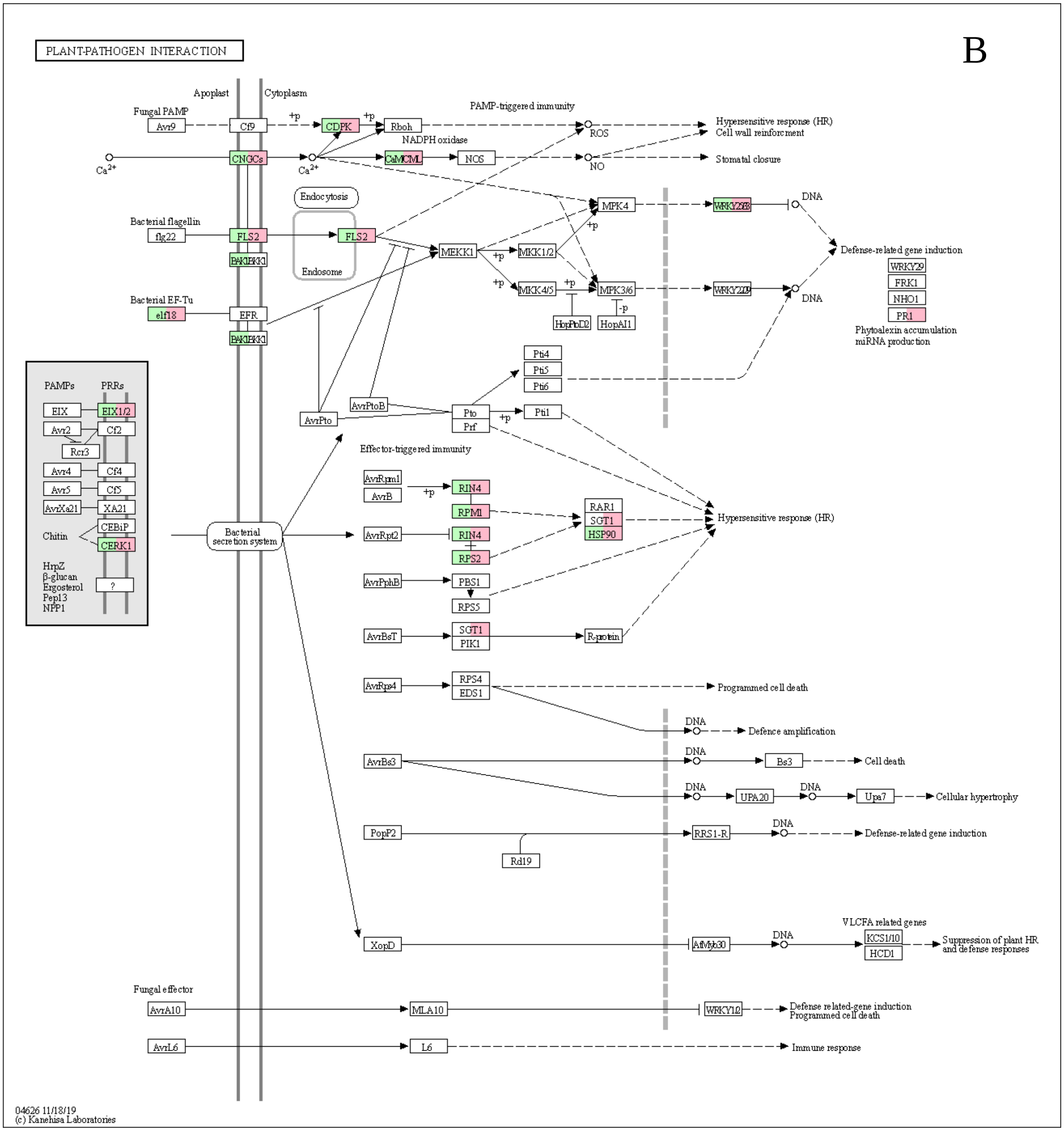

Supplementary figure S9. KEGG pathway analysis. Sequence data from the different treatments were assigned KO-numbers and mapped onto the MAPK signaling pathway - plant - Reference pathway (A) and the Plant-pathogen interaction - Reference pathway (B) to highlight similarities and differences between upregulated genes for the NBI vs NBNI (green) and the BI vs NBNI treatments.

Supplementary Table S6: List of *Fragaria vesca* and *Botrytis cinerea* primers used in this study.

Sl No	Primer Name	Primer Sequence (5'-3')	Gene IDs	Purpose
1	FvEF1aRTwF	GCCCATGGTTGTTGAAACTTT	FvH4_7g20050	qPCR
2	FvEF1aRTR	GGCGCATGTCCCTCACA		
3	FvPR1.1-RTF	CGGCGACTTATCAGGCACA	FvH4_2g02920	
4	FvPR1.1-RTR	CCACAAACCCTGCCAGAAGC		
5	FvPR5.3F	ACCTCCTAATGACACTCCCGAAACA	FvH4_6g16950	
6	FvPR5.3R	CGTAGTTAGGTCCACCGAAGCATGTA		
7	FvPR4-RTF	GCAGGACAACAACTGGGATTTG	FvH4_3g26010	
8	FvPR4-RTR	GTCACTAGCAGACATTTTCCACAGG		
9	FvBG2-1RTF	CCATATTGCTGCTCCTTGTTCTG	FvH4_3g28370	
10	FvBG2-1RTR	CCTTCCAATTCCATTGCTTTTGTAC		
11	FvBG2-3RTF	CCCTAATAAACAGCCAAAGTATCAGC	FvH4_4g19500	
12	FvBG2-3RTR	CGTATCACTCTTGAGAGAAGTGG		
13	FvPGIP1-RTF	CCTAGTTCATACGGGAAATTCGTTG	FvH4_6g22790	
14	FvPGIP1-RTR	TTCATGTTAGCAAATGAGGTTGGG		
15	FvLr10-JRTF	AACTCCTGCAGAGTATGCCAACTG	FvH4_4g26920	
16	FvLr10-RTR	GCGTTGCATGTGACCTCAAAC		

17	FvWRKY75-JRTF	CCCAGAAGCTACTATCGATGCAC	FvH4_6g53770	
18	FvWRKY75-RTR	AGGCTTGTCGATTGGATGAGAGTG		
19	FvCPK1-JRTF	TATGTCACCCTTGGGTTCAGGTTG	FvH4_6g20840	
20	FvCPK1-JRTR	GTGTTCAGCAATGACTCTAAGAGCC		
21	FvPecLy1-JRTF	GACATGAGTATCCGCAACAGCAC	FvH4_4g05760	
22	FvPecLy1-RTR	CACAACGCCAGCAATCATCAATG		
23	FvSLP3-JRTF	CTAGTGAATCAGTCACGTCCTATGC	FvH4_4g07370	
24	FvSLP3-JRTR	TTTCTGCCTGCTCGTGAGAAATG		
25	FvARR3-JRTF	GAATCTCCTCTTGCAAAGTGACCG	FvH4_4g35230	
26	FvARR3-JRTR	CCATCAAAACCAACTGAGGTGCTC		
27	FvPHOT2-JRTF	GAAACTGTCGGTTTCTTCAGGGAC	FvH4_2g17220	
28	FvPHOT2-JRTR	GTGAGAAGATTCCAGAAAGGAGTGC		
29	FvDSLPR-JRTF	GGCGGAGGATTTTAGTGACACATTG	FvH4_2g20290	
30	FvDSLPR-JRTR	CTATGCTTGCCTTGTGCTTGAGA		
31	FvPAL2-RTF	GAGGGCAGAGCTAGTAGAACATG	FvH4_7g19130	
32	FvPAL2-RTR	TTAGGCAAGACACTCTTCAGTTCC		
33	Fv4CL7-RTF	CAAAGACGACGTCGTCCTTATCC	FvH4_6g16460	
34	Fv4CL7-RTR	GATGACGAGCTTAGGGTTACAGTC		
35	FvGDS-RTF	AATGGTTGTAGTTAACGAGCCTGC		

36	FvGDS-RTR	GCAGTAGTGTAAAGGTCACCATCC	FvH4_4g27940	
37	FvBAS-RTF	AGCACAAACAACTTCGTCGGAAG	FvH4_2g08040	
38	FvBAS-RTR	GAACTGAAGACGAGCCTCTTCAAC		
39	FvSCMT-RTF	GAGAACCAGAGAGCTGTGATTTCG	FvH4_3g03130	
40	FvSCMT-RTR	TTCAGACACTCTGGGAAGAGAGTG		
41	Fv12OR2-RTF	GCATGGAAACCCATTGTCAATGC	FvH4_5g32630	
42	Fv12OR2-RTR	TCGGTACAAGAAATTGGAGCCTG		
43	FvACCOX1-RTF	GAGGTTCCAACCAACTATGACAGG	FvH4_5g19290	
44	FvACCOX1-RTR	CAGTTACTCCAGCATCAACAAGACC		
45	FvECDS-RTF	TTCTTCCCTCAATCTGCAATGCG	FvH4_2g23440	
46	FvECDS-RTR	TCCTTGGTAGGAAGAGCCTCTTC		
47	FvRe1 RTF	TGAATATGAAGAGGCTTGCAAAGACTC		
48	FvRe1 RTR	TCTTCTCCATCCTAATCCCATTCTCTG		
49	Bc3F	GCTGTAATTTCAATGTGCAGAATCC	(Diguta et al., 2010) For *B. cinerea* quantification	
50	Bc3R	GGAGCAACAATTAATCGCATTTC		
